# Self-Assembled Matrigel-Free iPSC-Derived Liver Organoids Demonstrate Wide-Ranging Highly Differentiated Liver Functions

**DOI:** 10.1093/stmcls/sxac090

**Published:** 2022-12-27

**Authors:** Yun Weng, Simon Han, Maria T Sekyi, Tao Su, Aras N Mattis, Tammy T Chang

**Affiliations:** Department of Surgery, University of California, San Francisco, CA 94143, USA; Department of Surgery, University of California, San Francisco, CA 94143, USA; Department of Surgery, University of California, San Francisco, CA 94143, USA; Department of Pathology, University of California, San Francisco, CA 94143, USA; Department of Pathology, University of California, San Francisco, CA 94143, USA; Liver Center, University of California, San Francisco, CA 94143, USA; Department of Surgery, University of California, San Francisco, CA 94143, USA; Liver Center, University of California, San Francisco, CA 94143, USA

**Keywords:** induced pluripotent stem cells, liver, organoids, RNA-Seq, Matrigel, self-assembly, microgravity

## Abstract

Human induced pluripotent stem cell (iPSC)-derived liver organoids serve as models of organogenesis, disease, drug screening, and regenerative medicine. Prevailing methods for generating organoids rely on Matrigel, whose batch-to-batch variability and xenogeneic source pose challenges to mechanistic research and translation to human clinical therapy. In this report, we demonstrate that self-assembled Matrigel-free iPSC-derived organoids developed in rotating wall vessels (RWVs) exhibit greater hepatocyte-specific functions than organoids formed on Matrigel. We show that RWVs produce highly functional liver organoids in part by eliminating the need for Matrigel, which has adverse effects on hepatic lineage differentiation. RWV liver organoids sustain durable function over long-term culture and express a range of mature functional genes at levels comparable to adult human liver, while retaining some fetal features. Our results indicate that RWVs provide a simple and high-throughput way to generate Matrigel-free liver organoids suitable for research and clinical applications.

## Graphical Abstract

**Graphical Abstract:**
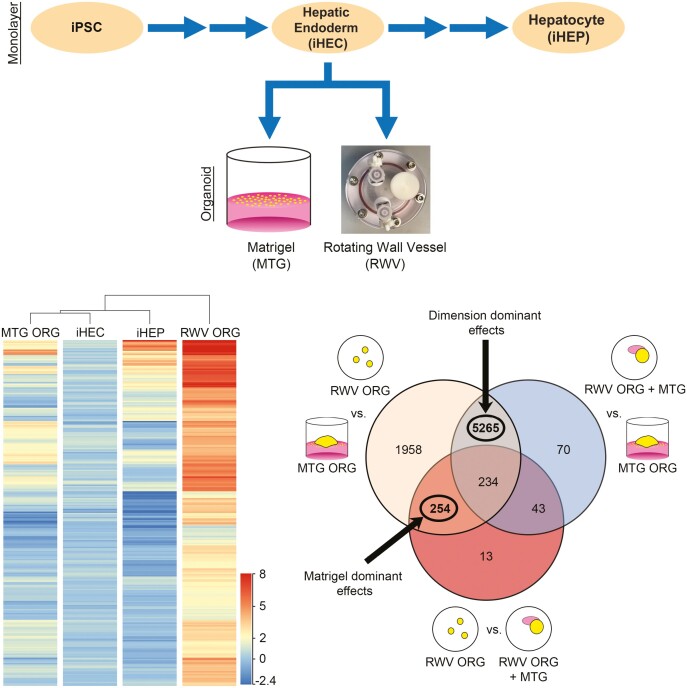

**Significance Statement**

Organoid self-assembly within rotating wall vessels (RWV) is a simple and high-throughput way to generate highly functional human induced pluripotent stem cell-derived liver organoids without the need for Matrigel. Liver organoids self-assembled in RWVs demonstrate higher, broader, and more durable hepatic function than organoids generated on Matrigel, in part because Matrigel has some adverse effects on hepatic lineage commitment.

## Introduction

Liver organoids are useful models to investigate organogenesis,^1^ hepatic disease,^2^ and drug metabolism.^3^ They have been used to support the function of liver-assist devices^4^ and have the potential to be implanted in patients as an alternative to organ transplant.^5-7^

The prevailing approach to generate liver organoids is to culture cells on Matrigel.^8-13^ Matrigel is a complex and incompletely defined mixture of extracellular matrix, growth factors, and signaling proteins derived from Engelbreth-Holm-Swarm mouse sarcoma cells. Proteomic profiling identified more than 1800 unique proteins in Matrigel, and batch-to-batch similarity was only 50%.^14^ This complexity and variability make it challenging to determine the factors that regulate three-dimensional (3D) organization and cell function in Matrigel-based organoids. In addition, the xenogeneic source of Matrigel is a barrier to using Matrigel-based organoids for clinical applications in human patients. Accordingly, there is increasing interest to develop Matrigel-independent organoid culture methods.^15^

We,^16,17^ and others,^18-21^ have shown that rotating wall vessels (RWV) produce hepatic organoids that have superior function compared to cell monolayers. Through solid-body rotation, RWVs provide a milieu with low turbulence, low shear stress, and 3D spatial freedom for cells to self-associate.^22^ These conditions simulate the properties of cell culture in microgravity, which has been explored as a unique environment for tissue engineering and biomanufacturing.^23-25^ We previously showed that primary hepatocytes within RWVs self-assembled into 100-200 μm-sized organoids, maintained 3D structure, and produced their own extracellular matrix.^7,17^

In this report, we compared the generation of human induced pluripotent stem cell (iPSC)-derived liver organoids on Matrigel versus in RWVs, and investigated the role of Matrigel in modulating hepatic differentiation. We hypothesized that Matrigel-free RWV-generated iPSC-derived organoids would exhibit improved form and function as compared to organoids generated on Matrigel.

## Material and Methods

### Hepatic Organoid Generation from Induced Hepatic Endoderm Cells (iHEC)

A derivative of the reference human iPSC line Wt11c, which was extensively characterized to be pluripotent and genetically stable,^26^ was obtained from Coriell Institute (Camden, NJ) and maintained with Cultrex basement membrane extract (R&D Systems, Minneapolis, MN) and StemFlex media (Thermo Fisher Scientific, Waltham, MA). Differentiation toward the hepatic lineage was initiated as monolayers on 30-fold-diluted Matrigel (Corning Life Science, Glendale, AZ) using established protocols^27,28^ and described in more detail in Supplemental Methods. To generate iHEC-derived organoids, cells were released by Accutase (MilliporeSigma, St. Louis, MO) and resuspended in Organoid Media that consisted of 50% HCM media (Lonza, Walkersville, MD) and 50% EBM2 media (Lonza) with 40 ng/mL HGF (PeproTech, Cranbury, NJ), 20 ng/mL OSM (PeproTech) and 1 μg/mL dexamethasone (Thomas Scientific, Swedesboro, NJ).^29^ Cells were seeded either onto 24-well-plates coated with 2-fold-diluted Matrigel, or into 10 mL RWVs (Synthecon, Houston, TX) set to 10.5 rpm rotation. For RWV organoid plus Matrigel (RWV ORG + MTG) conditions, 50 μL of 2-fold-diluted Matrigel was added to RWVs at the start of culture. All organoid cultures were incubated in 37 ^o^C with 20% O_2_ and 5% CO_2_. Cell densities for all conditions were 5 × 10^5^/mL, and media were changed every 2 days.

### Statistics

RNA-seq differential gene expression, clustering, and statistical analyses were performed using GeneSpring GX v14.9 (Agilent Technologies, Santa Clara, CA). Additional statistical analyses were performed with Prism v9.0.0 (GraphPad, La Jolla, CA). Details regarding statistical tests and parameters used for analysis in each figure are reported in the corresponding figure legend.

Additional methodological details are provided in Supplemental Methods.

## Results

### Organoids Generated in RWVs from iHECs Demonstrate Greater Expression of Key Hepatic Functional Genes Compared to Organoids Generated on Matrigel

**Figure 1.**
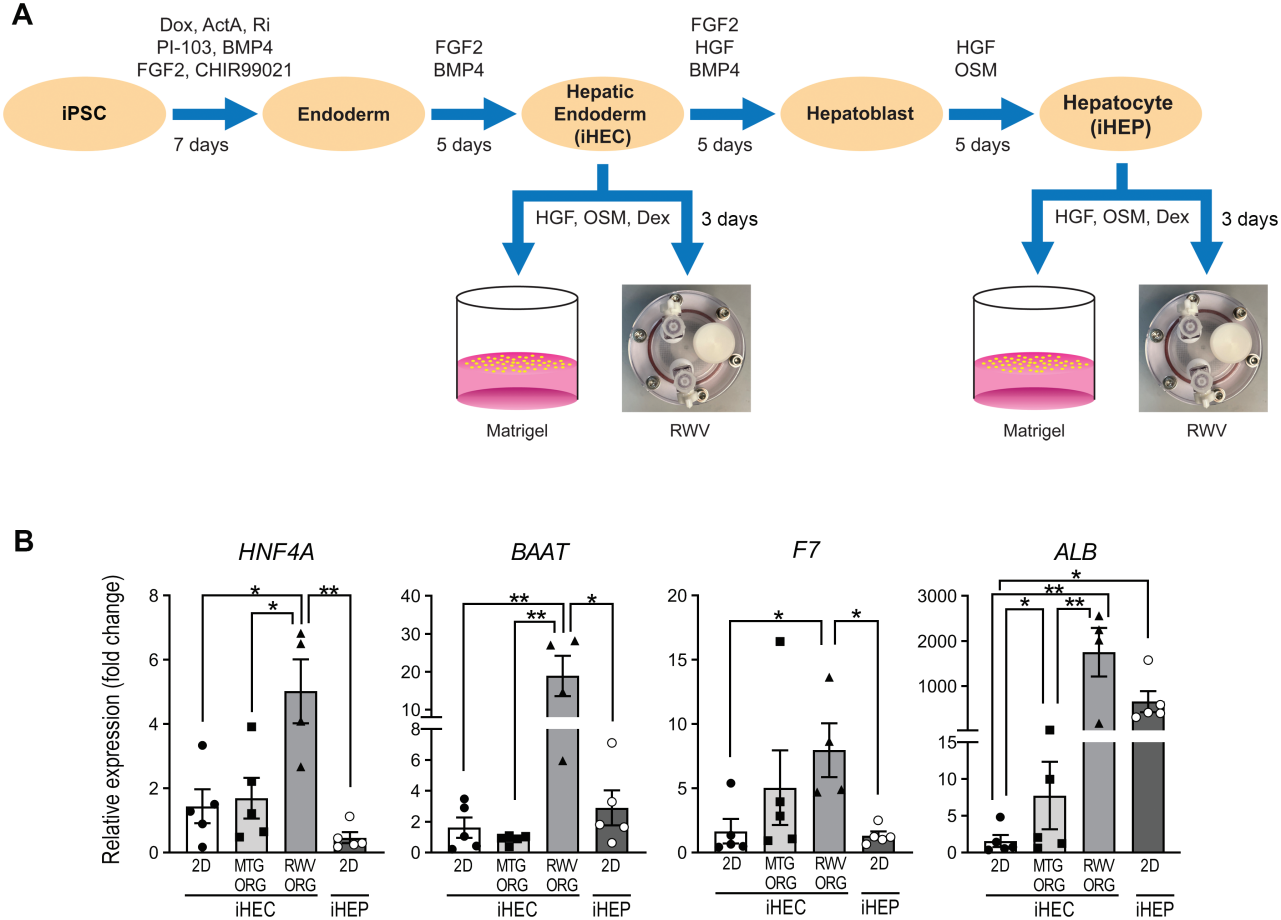
RWV organoids generated from induced hepatic endoderm cells (iHECs) demonstrate greater expression of key hepatic functional genes compared to Matrigel (MTG) organoids generated from iHECs and induced hepatocyte (iHEP) monolayers. (**A**) Experimental protocol of human iPSC differentiation and hepatic organoid generation. Dox, doxycycline; ActA, activin A; Ri, ROCK inhibitor Y27632; Dex, dexamethasone. (**B**) Quantitative real-time reverse transcription PCR (qRT-PCR) analysis showing gene expression of iHEC monolayers (iHEC 2D), iHEC-derived MTG ORGs and RWV ORGs after 3 days of organoid culture, and iHEP monolayers (iHEP 2D), represented as relative fold change compared to iHEC 2D. Data show independent biological samples and mean ± SEM; *P < .05; **P < .01 by 2-tailed Student’s *t* test.

We used standard established protocols to differentiate human iPSCs toward the hepatic lineage^27,28^ and generated organoids from the iHEC and hepatocyte (iHEP) stages (Fig. 1A). After 3 days of organoid culture on Matrigel or in RWVs, we compared the gene expression of organoids with iHEC and iHEP two-dimensional (2D) monolayers. RWV organoids generated from iHECs (RWV ORGs) showed significantly greater expression of hepatocyte nuclear factor 4 alpha (*HNF4A*), a master transcriptional regulator of hepatocyte function,^30,31^ as compared to iHEC monolayers, iHEC Matrigel organoids (MTG ORGs), and iHEP monolayers (Fig. 1B). Similarly, direct transcriptional targets of *HNF4A*, such as *BAAT* and *F7* (examples of liver metabolic and synthetic functions, respectively) were also more highly expressed in iHEC RWV ORGs. iHEC RWV ORGs and monolayer iHEPs both showed markedly upregulated expression of *ALB*, a common benchmark of hepatic function, as compared to iHEC monolayers and iHEC MTG ORGs. Thus, organoid formation in RWVs at the iHEC stage improved hepatocyte-specific functional gene expression and accelerated hepatocyte maturation by 7 days compared to monolayer differentiation. In contrast, organoids generated from iHEP monolayers showed downregulated hepatic functional gene expression as compared to the originating monolayer iHEP cells (Supplemental Fig. S1). These results suggest that self-assembly of organoids in RWVs from iHECs accelerates and improves differentiation toward mature hepatocyte function, but organoid formation after the iHEP stage of differentiation adversely affects hepatic function.

Our research subsequently focused on RWV and Matrigel organoids generated from iHECs. Accordingly, in this report, we use RWV ORGs and MTG ORGs as abbreviations for RWV and Matrigel organoids generated from iHECs.

### RNA Sequencing Analysis Shows That RWV ORGs Resemble the Hepatic Lineage More Closely than MTG ORGs or iHEP Monolayers

**Figure 2.**
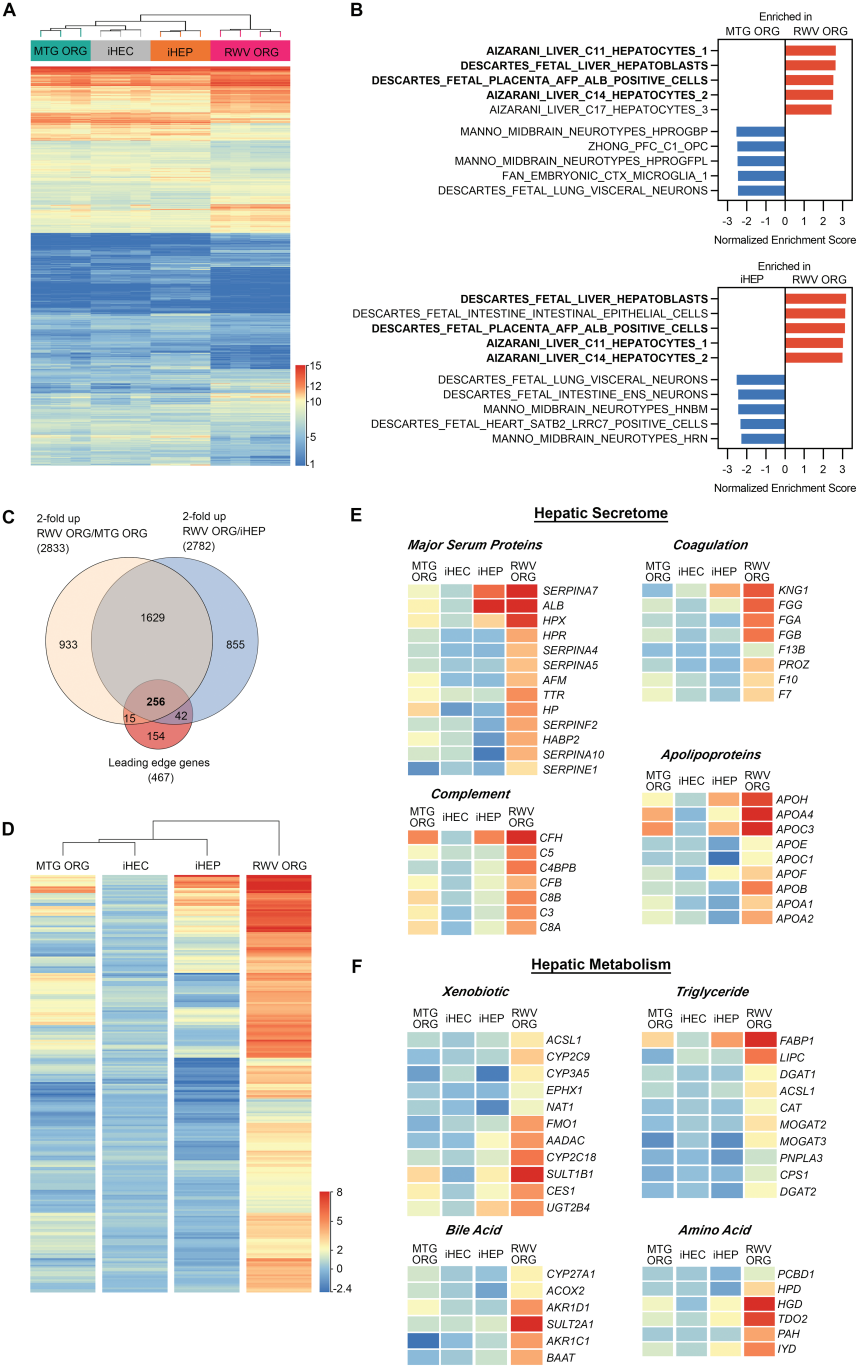
Organoids generated in RWVs from iHECs (RWV ORG) differentiate closely toward hepatocytes and hepatoblasts, whereas Matrigel organoids generated from iHECs (MTG ORG) and iHEP monolayers are enriched in other cell types. (**A**) RNA-seq heatmap and hierarchical clustering of 8955 significantly differentially expressed genes as determined by one-way ANOVA comparing the 4 conditions. Each lane represents an independent biological sample from separate differentiation experiments (*n* = 3-4). Gene expression represents log_2_ DESeq2 normalized counts. (**B**) Gene set enrichment analysis (GSEA) was performed using cell-type-signature gene sets curated from single-cell sequencing data of human tissues. All gene set enrichment *P* < .001 and false discovery rate (FDR) < .001. Bolded gene sets are enriched in RWV ORG compared to both MTG ORG and iHEPs. (**C**) Venn diagram of genes significantly upregulated by ≥2-fold in RWV ORGs compared to MTG ORGs or iHEP monolayers, and hepatic lineage genes as determine by GSEA Leading Edge Analysis. (**D**) Heatmap and hierarchical clustering of 256 hepatic-enriched genes significantly upregulated by ≥2-fold in RWV ORGs. Each lane represents the average expression of 3-4 independent biological samples. Expression levels are shown as log_2_ DESeq2 normalized gene expression scaled to iHEC monolayer expression levels as baseline. (**E**) Major classes of hepatic functional genes significantly upregulated in RWV ORGs. Expression level color legend is the same as in (**D**).

To characterize the functional capabilities of RWV ORGs in detail, we performed bulk RNA-sequencing (RNA-seq) on iHEC monolayers, MTG ORGs and RWV ORGs derived from iHECs after 3 days of organoid culture, and iHEP monolayers. Comparing the 4 conditions, one-way ANOVA identified 8955 genes significantly differentially expressed ≥2-fold between at least 1 pair of conditions (Supplemental Table S1). Unsupervised hierarchical clustering analysis indicated that iHEC monolayers, iHEC-derived MTG ORGs, and iHEP monolayers clustered together, whereas iHEC-derived RWV ORGs clustered under its own branch separately from the 3 other conditions (Fig. 2A).

To determine how organoid culture conditions may regulate cell fate, we conducted Gene Set Enrichment Analysis (GSEA) using the cell-type-signature gene set collection, which contained 700 gene sets representing marker genes for cell types identified in single-cell sequencing of human tissues. We found that gene sets representing human mature hepatocytes and fetal hepatoblasts were significantly enriched in RWV ORGs as compared to both MTG ORGs and iHEPs (Fig. 2B, Supplemental Tables S2 and S3). Top gene sets enriched in RWV ORGs included major mature hepatocyte populations identified by single-cell sequencing of normal adult human liver,^32^ as well as gene sets representing fetal hepatoblasts and placental cells expressing major hepatoblast markers (*ALB* and *AFP*).^33^ In contrast, gene sets enriched in MTG ORGs and iHEP monolayers demonstrated non-liver cells, with a predominance of neuronal cell types (Fig. 2B, Supplemental Tables S4 and S5). Our results suggest that RWV ORG culture conditions enhance the differentiation of iPSCs toward the hepatic lineage, whereas MTG ORG and iHEP monolayer conditions support differentiation towards other cell types, including toward the neuronal cell fate.

GSEA Leading Edge Analysis of Fig. 2B gene sets enriched in RWV ORGs yielded 467 genes that were responsible for the core enrichment of hepatocyte/hepatoblast cell type signature gene sets in the RWV ORG condition. Differential gene expression analysis identified 2833 genes that were significantly upregulated by ≥2-fold in RWV ORGs compared to MTG ORGs, and 2782 genes that were upregulated by ≥2-fold in RWV ORGs compared to iHEP monolayers. Venn diagram intersection of these 3 gene sets showed 256 genes that represent the most highly upregulated hepatic-enriched genes in RWV ORGs (Fig. 2C and Supplemental Table S6).

Using iHEC monolayers as the baseline from which the differentiation milieu diverged into the MTG ORG, RWV ORG, and iHEP monolayer conditions, we performed clustering analysis on the 256 highly differentially regulated genes (Fig. 2D). We found that RWV ORGs upregulated the expression of these 256 genes significantly as compared to iHEC monolayer baseline, whereas MTG ORGs and iHEP monolayers upregulated a minority of these genes (<50%), and the magnitude of upregulation was less than that demonstrated by RWV ORGs.

Importantly, the 256 hepatic-enriched genes highly upregulated in RWV ORGs represented broad and diverse categories of mature hepatocyte function. The synthesis and secretion of proteins essential to various physiological systems is a major energy-intensive function of hepatocytes.^34^ We found that the hepatic secretome, encompassing multiple and diverse classes of secreted proteins, was upregulated in RWV ORGs as compared to MTG ORGs and iHEP monolayers (Fig. 2E). Similarly, RWV ORGs significantly upregulated expression of genes critical to key hepatocyte-specific metabolic processes, including xenobiotic, bile acid, triglyceride, and amino acid metabolic pathways (Fig. 2F). These findings indicate that RWV ORG culture conditions promote higher-fidelity differentiation toward the hepatic lineage and broadly enhance the expression of a wide range of hepatocyte functional genes.

### Hepatic Differentiated Functions Are Upregulated in RWV ORGs, Whereas Proliferation Is Upregulated in MTG ORGs

**Figure 3.**
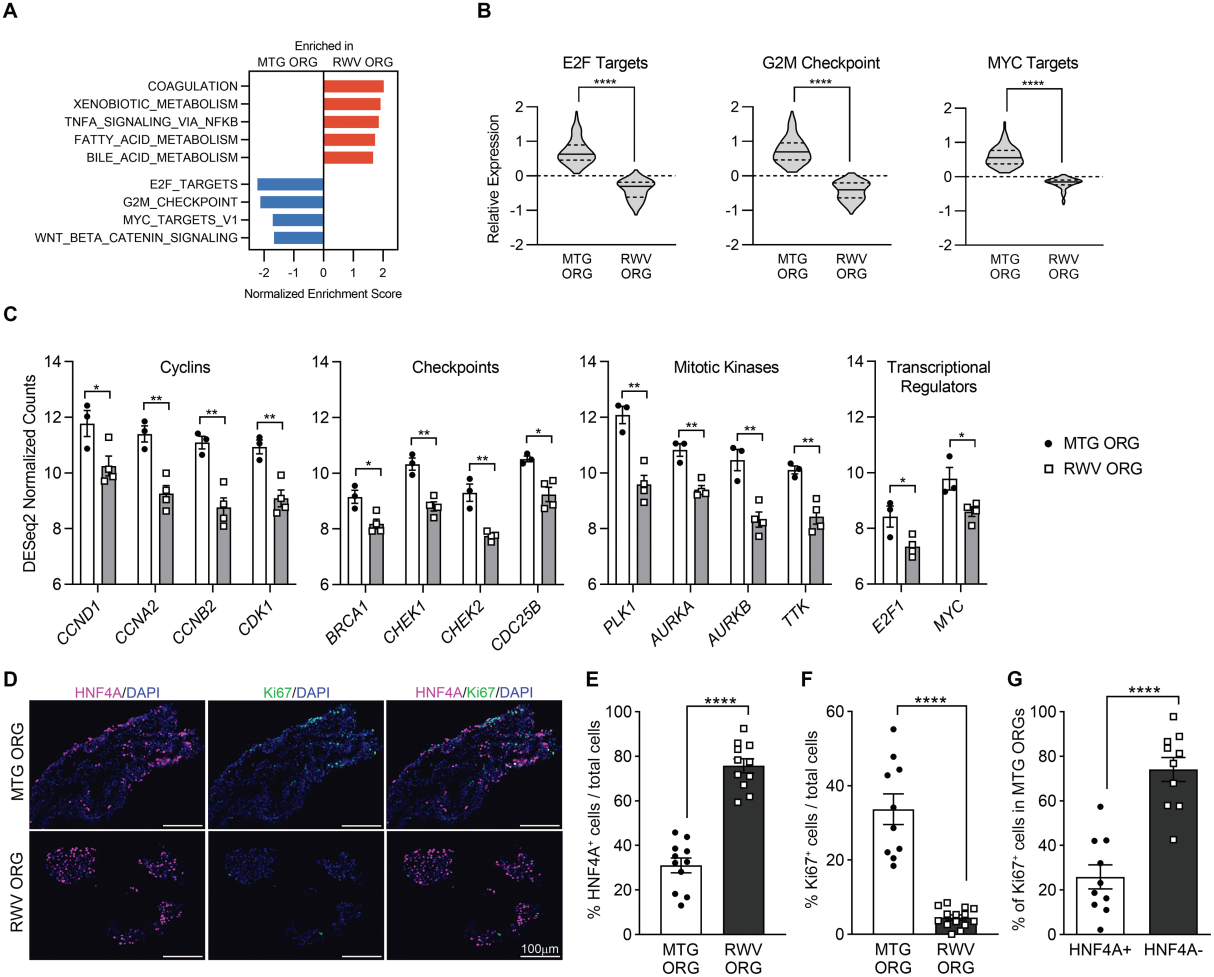
Proliferation is increased in MTG ORGs as compared to RWV ORGs, and proliferating cells in MTG ORGs are predominantly HNF4A-negative. (**A**) Hallmark gene sets enriched in RWV ORGs and MTG ORGs as determined by GSEA; all gene set enrichment *P* < .001, FDR < .01. (**B**) Violin plots show the expression distribution of leading-edge genes in the E2F targets (147 genes), G2/M checkpoint (130 genes), and MYC targets (130 genes) gene sets. Relative expression represents the average of 3-4 independent biological samples and shows log_2_ DESeq2 normalized counts scaled to the median of all samples. Solid line within the violin plots shows median and dotted lines show quartiles. ****P < .0001 by 2-tailed paired *t* test. (**C**) Key cell cycle progression and proliferation genes significantly upregulated in MTG ORGs as compared to RWV ORGs. Data points represent individual biological samples and bars show average ± SEM. *P < .05, **P < .01 by 2-tailed Student’s *t* test. (**D**) Immunostaining of MTG ORGs and RWV ORGs after 4 days of organoid culture showing HNF4A, Ki67, and DAPI. Scale bar = 100 µm. Images are representative of organoids analyzed from 3 independent experiments. (**E**) Percentage of HNF4A^+^ cells of total cells in MTG ORGs and RWV ORGs. Numbers of HNF4A^+^ cells and total DAPI^+^ cells were counted in each fluorescence image, and % of HNF4A^+^ cells calculated as (number of HNF4A^*+*^ cells/ number of total cells) * 100. (**F**) Percentage of Ki67^+^ cells of total cells in MTG ORGs and RWV ORGs. Numbers of Ki67^+^ cells and total DAPI^+^ cells were counted in each fluorescence image, and % of Ki67^+^ cells calculated as (number of Ki67^*+*^ cells/number of total cells) * 100. (**G**) HNF4A expression among Ki67^+^ cells in MTG ORGs. Numbers of HNF4A^+^Ki67^+^ or HNF4A^−^Ki67^+^ cells were determined and divided by the total number of Ki67^+^ cells in each fluorescence image to obtain percentages. For (**E**), (**F**), and (**G**) low-powered images (20× magnification) were taken of each condition from 3 independent experiments. Graphs show data from individual images and mean ± SEM; ****P < .0001 by 2-tailed Student’s *t* test.

We performed GSEA to compare MTG ORGs and RWV ORGs using the hallmark gene set, which represented specific well-defined biological processes and genes that displayed coordinated expression. The most highly enriched gene sets in RWV ORGs included coagulation, xenobiotic metabolism, fatty acid metabolism, and bile acid metabolism (Fig. 3A and Supplemental Table S7), consistent with the upregulated functional genes we identified through cell-type-signature analysis (Fig. 2E, 2F). In addition to pathways related to hepatocyte function, the tumor necrosis factor alpha (TNFA) signaling pathway was also significantly enriched in RWV ORGs. Conversely, MTG ORGs demonstrated enrichment in E2F targets, G2/M cell cycle checkpoints, and MYC targets, suggesting increased cell cycle progression and proliferation. The WNT/β-catenin pathway was also moderately enriched in MTG ORGs (Fig. 3A and Supplemental Table S8).

To further investigate the differential expression of proliferative markers between the two organoid culture conditions, we examined the leading-edge genes identified by GSEA to be most responsible for the enrichment of E2F targets, G2/M checkpoints, and MYC targets in MTG ORGs (Fig. 3B, Supplemental Tables S9–S11). Upregulated genes in MTG ORGs included key cyclins, DNA damage checkpoint regulators, and mitotic kinases (Fig. 3C). *E2F1* and *MYC*, apex transcriptional regulators of cell cycle entry and proliferation, were themselves more highly expressed in MTG ORGs as compared to RWV ORGs. These findings indicated that all phases of the cell cycle were more activated in MTG ORGs than in RWV ORGs.

To determine the characteristics of proliferating cells in MTG ORGs, we performed immunohistochemistry using HNF4A to identify hepatic lineage cells and Ki67 to identify proliferating cells (Fig. 3D). Consistent with gene expression data, RWV ORGs demonstrated more HNF4A^+^ cells than MTG ORGs, whereas MTG ORGs demonstrated more Ki67^+^ cells than RWV ORGs (Fig. 3D–3F). Among proliferating Ki67^+^ cells in MTG ORGs, the majority (~75%) were HNF4A^−^ cells, which either represented non-hepatic lineage cells or hepatic cells that lost HNF4A expression (Fig. 3D, 3G). Previous studies have suggested that elaboration of differentiated functions and proliferation are often divergent pathways.^35^ Our results indicate that RWV ORG culture conditions promote greater hepatocyte differentiated function, whereas MTG ORG conditions permit more proliferation predominantly in cells with non-hepatic features.

### Genes That Regulate Tissue Structure Are Significantly more Highly Expressed in RWV ORGs Than MTG ORGs

**Figure 4.**
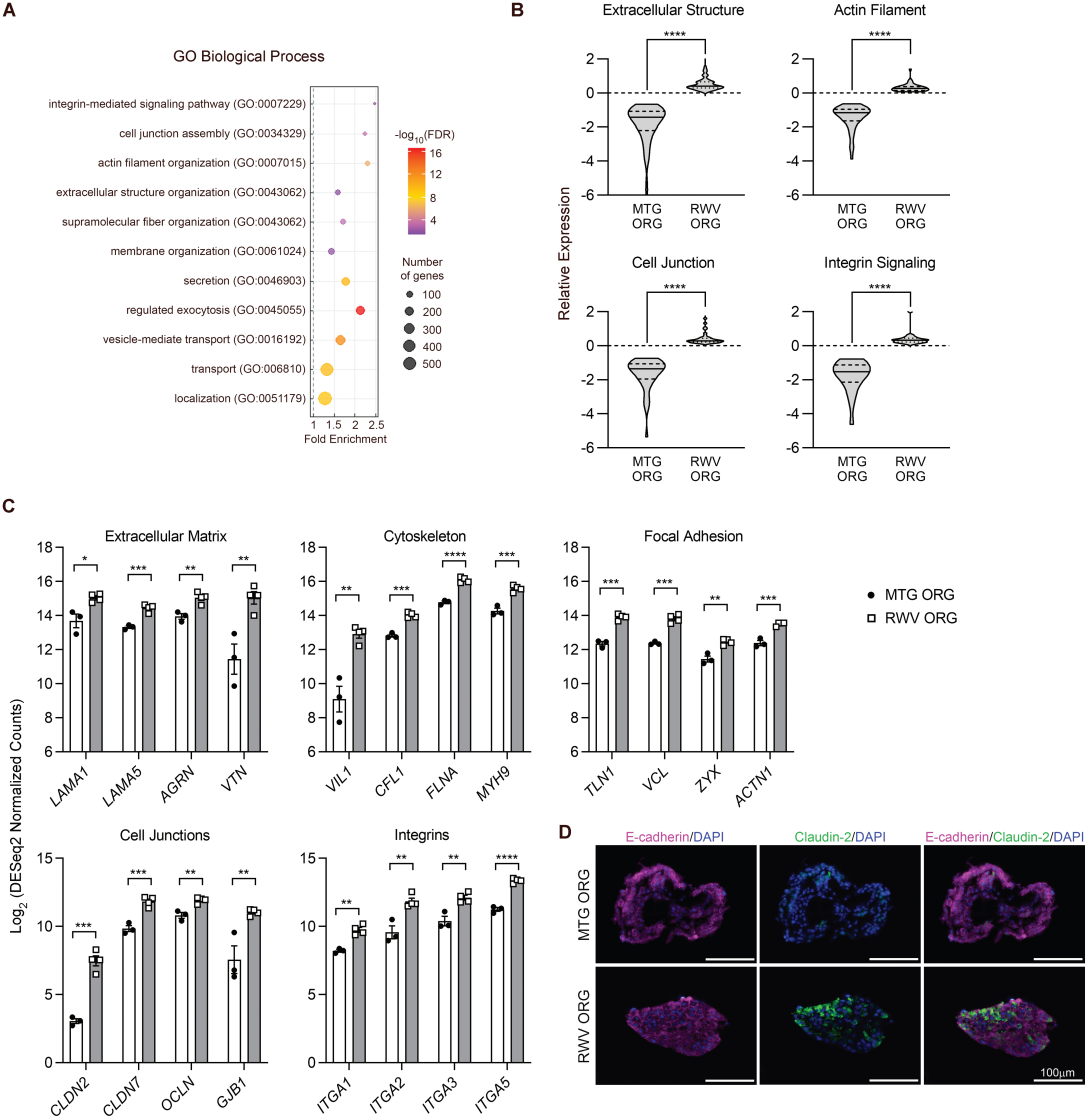
Cell and tissue structure genes are upregulated in RWV ORGs and correlate with the presence of claudin-2 positive cells, suggesting increased cell–cell adhesion complexity. (**A**) Biological process gene ontology (GO) terms significantly enriched in genes ≥2-fold upregulated in RWV ORGs as compared to MTG ORGs. Color gradient represents −log_10_ false discovery rate (FDR). Size of circles represents the number of genes in the term that are ≥2-fold upregulated in RWV ORGs. (**B**) Violin plots show the expression distribution of genes encompassed in the biological process GO terms: extracellular structure organization (58 genes), actin filament organization (36 genes), cell junction assembly (36 genes), and integrin-mediated signaling pathway (20 genes). Relative expression represents the average of 3-4 independent biological samples and shows log_2_ DESeq2 normalized counts scaled to the median of all samples. Solid line within the violin plots shows median and dotted lines show quartiles. ****P < .0001 by 2-tailed paired *t* test. (**C**) Key cell and tissue structure genes significantly upregulated in RWV ORGs as compared to MTG ORGs. Data points represent individual biological samples and bars show average ± SEM. *P < .05, **P < .01, ***P < .001, and ****P < .0001 by 2-tailed Student’s *t* test. (**D**) Immunostaining of MTG ORGs and RWV ORGs after 4 days of organoid culture showing E-cadherin , claudin-2, and DAPI. Scale bar = 100 µm. Images are representative of organoids analyzed from 3 independent experiments.

To identify biological processes associated with, and potentially underpinning, augmented hepatocyte-specific functions in RWV ORGs, we performed Gene Ontology (GO) Analysis on genes ≥2-fold upregulated in RWV ORGs as compared to MTG ORGs. This analysis yielded 301 biological process terms that were significantly enriched (threshold *P*-value < .001) (Supplemental Table S12). GO Analysis revealed that membrane-related processes such as vesicle-mediated transport, exocytosis, and secretion were significantly upregulated in RWV ORGs (Fig. 4A). Moreover, processes that regulated tissue structure through cytoskeletal organization, cell–cell adhesion, and cell–matrix interactions were highly enriched in RWV ORGs as compared to MTG ORGs.

We next analyzed the genes that were identified through the GO terms: extracellular structure organization, actin filament organization, cell junction assembly, and integrin-mediated signaling pathway (Fig. 4B, Supplemental Tables S13–S16). We found that key genes encoding basement membrane laminins (*LAMA1* and *LAMA5*), proteoglycans (*AGRN*), and adhesive matricellular glycoproteins (*VTN*) were significantly more highly expressed in RWV ORGs than MTG ORGs (Fig. 4C). Major cytoskeletal regulators, including those that modulate actin filament capping (*VIL1*), polymerization (*CFL1*), cross-linking (*FLNA*), and contractility (*MYH9*), were significantly upregulated in RWV ORGs. Moreover, the expression of important components of the focal adhesion complex (*TLN1*, *VCL*, *ZYX*, and *ACTN1*), which link the cytoskeleton to the cell membrane and extracellular matrix, were also increased in RWV ORGs. RWV ORGs expressed greater levels of genes encoding tight junction (*CLDN2*, *CLDN7*, *OCLN*) and gap junction (*GJB1*) molecules, which mediate cell–cell adhesion and communication, as compared to MTG ORGs. RWV ORGs also demonstrated increased expression of integrin subunits that bind to all major species of extracellular matrix proteins found in normal liver: *ITGA1*—collagen IV, *ITGA2*—collagen I, *ITGA3*—laminin, and *ITGA5*—fibronectin. These findings show that the entire machinery needed to build tissues from cells, by linking the interior cytoskeleton of cells with other cells and surrounding matrix, is upregulated in RWV ORGs as compared to MTG ORGs.

E-cadherin is a critical component of adherens junctions and plays important roles in maintaining epithelial cell identity. Hepatic organoids have shown generalized expression of E-cadherin,^9,10^ whereas in liver tissue, E-cadherin is predominantly expressed by peri-portal hepatocytes.^36^ Our data show that RWV ORGs markedly upregulate the gene expression of claudin-2 (*CLDN2*) (Fig. 4C), which encodes a tight junction protein highly expressed by perivenous hepatocytes in the adult liver.^37^ We performed immunostaining for E-cadherin and claudin-2 to determine the localization of these 2 cell–cell adhesion molecules. Whereas E-cadherin was diffusely expressed in both MTG ORGs and RWV ORGs, claudin-2 expression was evident in some cells within RWV ORGs and not detected in MTG ORGs (Fig. 4D). These results suggest that enhanced expression of hepatic functional genes in RWV ORGs may correlate with the increased complexity of the cell–cell adhesion apparatus within the organoids.

### Hepatic Function in MTG ORGs Is Lower Partly Because Matrigel Promotes Alternative Differentiation Pathways

**Figure 5.**
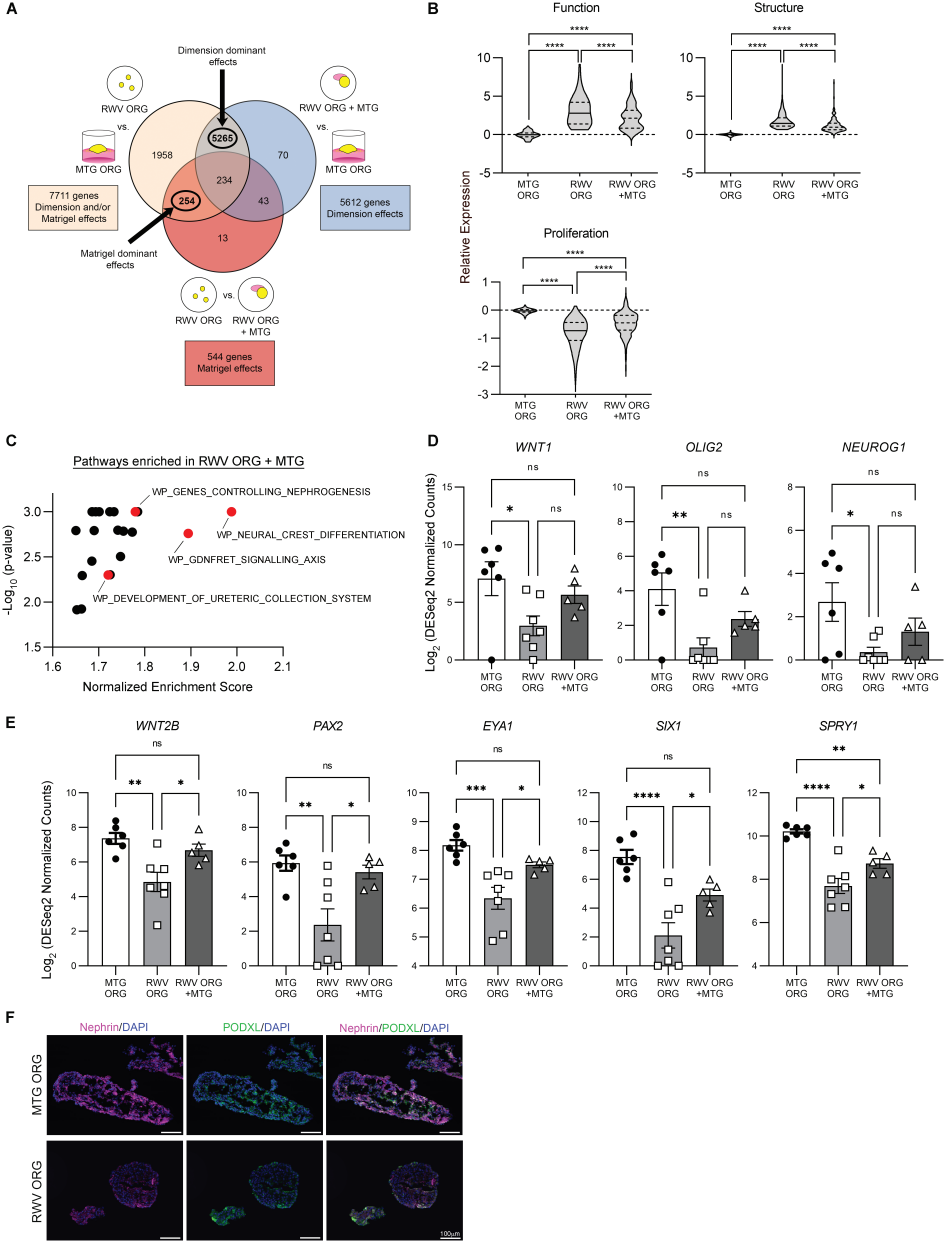
Enhanced hepatic function and structure in RWV ORGs are primarily attributable to Dimension-dominant milieu effects, whereas Matrigel-dominant effects include induction of genes that promote nephrogenesis. (**A**) Venn diagram depicting the RNA-seq differential gene expression analysis scheme and the combinations of comparisons made between the 3 conditions: MTG ORG, RWV ORG, and RWV ORG + MTG. (**B**) Violin plots show the expression distribution of genes in the combined hepatic function (69 genes), tissue structure (142 genes), and proliferation (316 genes) gene lists. Relative expression represents the average of 3-4 independent biological samples and shows log_2_ DESeq2 normalized counts scaled to the median of MTG ORG samples. Solid line within the violin plots shows median and dotted lines show quartiles. ****P < .0001 by one-way repeated measures ANOVA and post-hoc Tukey’s HSD. (**C**) Curated canonical pathway gene sets significantly enriched in RWV ORG + MTG as compared to RWV ORG as determined by GSEA with FDR<25%. Each point represents a unique gene set. Select gene sets are highlighted and annotated; *WP*, WikiPathways. (**D**) Key genes important in neural crest development upregulated by the presence of Matrigel. (**E**) Key genes important in kidney development significantly upregulated by the presence of Matrigel. For (**D**) and (**E**), data points represent individual biological samples and bars show average ± SEM. *P < .05, **P < .01, ***P < .001, ****P < .0001, and ns = not significant, by one-way ANOVA and post-hoc Tukey’s HSD. (**F**) Immunostaining of MTG ORGs and RWV ORGs after 4 days of organoid culture showing nephrin (NPHS1), POXDL, and DAPI. Scale bar = 100 µm. Images are representative of organoids analyzed from 3 independent experiments.

Functional differences between MTG ORGs and RWV ORGs may be modulated by the presence or absence of Matrigel. Combinatorial effects of the different culture milieus may also play a role. Key distinctions include that MTG ORGs develop from flat 2D surfaces in static culture, whereas RWV ORGs self-assemble in dynamic rotational 3D culture. To explore the contribution of these factors in modulating cell fate differentiation and function, we added Matrigel as a carrier for organoid formation within RWVs. We found that whereas numerous 100-200 μm-diameter organoids developed in standard RWV ORG conditions, the addition of 50 μL of Matrigel in RWVs resulted in the formation of a few 500-1000 μm-diameter organoids. We then performed RNA-seq differential gene expression analysis on MTG ORG, RWV ORG, and RWV ORG + MTG conditions (Fig. 5A). We denoted the combinatorial effects of 2D static versus 3D dynamic conditions as “Dimension” variables that could be partially isolated by comparing MTG ORG versus RWV ORG + MTG conditions. We denoted “Matrigel effects” as gene expression differences attributable to the presence or absence of Matrigel that may be determined by comparing RWV ORG versus RWV ORG + MTG conditions.

RNA-seq analysis of the 3 conditions yielded 7711 genes that were significantly differentially expressed between RWV ORG and MTG ORG conditions; these differences could be primarily regulated by Dimension effects, Matrigel effects, or interactions of Dimension and Matrigel effects (Fig. 5A). Comparison of RWV ORG + MTG and MTG ORG conditions resulted in 5612 differentially expressed genes that were primarily attributable to differences in Dimension factors. RWV ORG versus RWV ORG + MTG analysis showed 544 genes that were chiefly modulated by the presence or absence of Matrigel. A Venn diagram of these sets of differentially expressed genes demonstrated 5265 genes at the intersection of “RWV ORG vs. MTG ORG” and “RWV ORG + MTG vs. MTG ORG” gene sets; these differences were interpreted as the results of Dimension-dominant effects. Conversely, the intersection of “RWV ORG vs. MTG ORG” and “RWV ORG vs. RWV ORG + MTG” gene sets showed 254 genes, which were interpreted as the results of Matrigel-dominant effects. Thus, the majority of the gene expression differences between MTG ORGs and RWV ORGs may be accounted for by Dimension-dominant effects (5265/7711 genes = 68%), whereas as a small fraction might be explained by Matrigel-dominant effects (254/7711 genes = 3%).

We used differentially regulated genes in hepatic function, proliferation, and tissue structure as readouts to examine the RWV ORG + MTG condition (Fig. 5B, Supplemental Tables S17–S19). For hepatic function, we compiled all the genes that were characterized in Fig. 2E, 2F into a single gene list. Similarly, all the genes represented in Fig. 3B were compiled into a master proliferation gene list, and all the genes described in Fig. 4B were condensed into a total tissue structure gene list. We found that hepatic function genes were significantly upregulated in RWV ORG and RWV ORG + MTG conditions in comparison with MTG ORG (Fig. 5B). RWV ORG + MTG showed modest-magnitude statistically significant reduced functional gene expression as compared to RWV ORG. Tissue structure genes demonstrated a similar expression pattern between the 3 conditions. Proliferation genes followed the reverse pattern in which expression was decreased in RWV ORG and RWV ORG + MTG conditions as compared to MTG ORG, and slightly increased in RWV ORG + MTG compared to RWV ORG. These analyses are consistent with a paradigm in which phenotypic differences between MTG ORGs and RWV ORGs are predominantly determined by Dimension effects, and the presence of Matrigel has some modulating impact.

To determine which pathways might be preferentially induced by the presence of Matrigel, we performed GSEA using the curated canonical pathways gene sets to identify pathways enriched in RWV ORG + MTG as compared to RWV ORG. This analysis revealed 22 gene sets that had false discovery rates of <25% (Fig. 5C and Supplemental Table S20). The most highly enriched statistically significant pathway in RWV ORG + MTG was neural crest differentiation, suggesting that Matrigel has a role in promoting neural cell fates. *WNT1*, *OLIG2*, and *NEUROG1*, important markers of neural crest differentiation, showed significantly higher expression in MTG ORGs as compared to RWV ORGs, and exhibited a trend toward increased expression in the RWV ORG + MTG condition compared to RWV ORGs (Fig. 5D). In addition, 3 pathways with overlapping functions in kidney organogenesis (glial cell line-derived neurotrophic factor [GDNF]/RET tyrosine kinase signaling axis, genes controlling nephrogenesis, and development of ureteric collection system) were significantly enriched in RWV ORG + MTG as compared to RWV ORG. Key members of these pathways included *WNT2B*, *PAX2*, *EYA1*, *SIX1*, and *SPRY1*. Each of these genes showed significantly less expression in RWV ORGs as compared to MTG ORGs, and addition of Matrigel increased gene expression in RWV ORG + MTG conditions to levels comparable to those in MTG ORGs (Fig. 5E). In contrast to the liver, which develops from the endoderm, kidneys develop from the intermediate mesoderm. *WNT2B* has been implicated in both liver and kidney development, and its role may be context dependent.^38^*PAX2*, *EYA1*, *SIX1*, and *SPRY1* are genes critical in regulating kidney development and have no known roles in liver organogenesis.^39^ Immunohistochemical staining for nephrin (NPHS1) and PODXL, two markers of nephrogenic differentiation, showed greater expression in MTG ORGs than in RWV ORGs (Fig. 5F), corroborating with gene expression data that Matrigel promoted non-hepatic differentiation pathways. These results suggest that Matrigel plays a role in inducing alternative differentiation pathways, including neurogenic and nephrogenic programs, which likely contribute to reduced hepatic function in MTG ORGs.

### RWV ORGs Demonstrate Stable Organoid Structure and Maintain Greater Hepatocyte-Specific Functions as Compared to MTG ORGs Over Long-Term Culture

**Figure 6.**
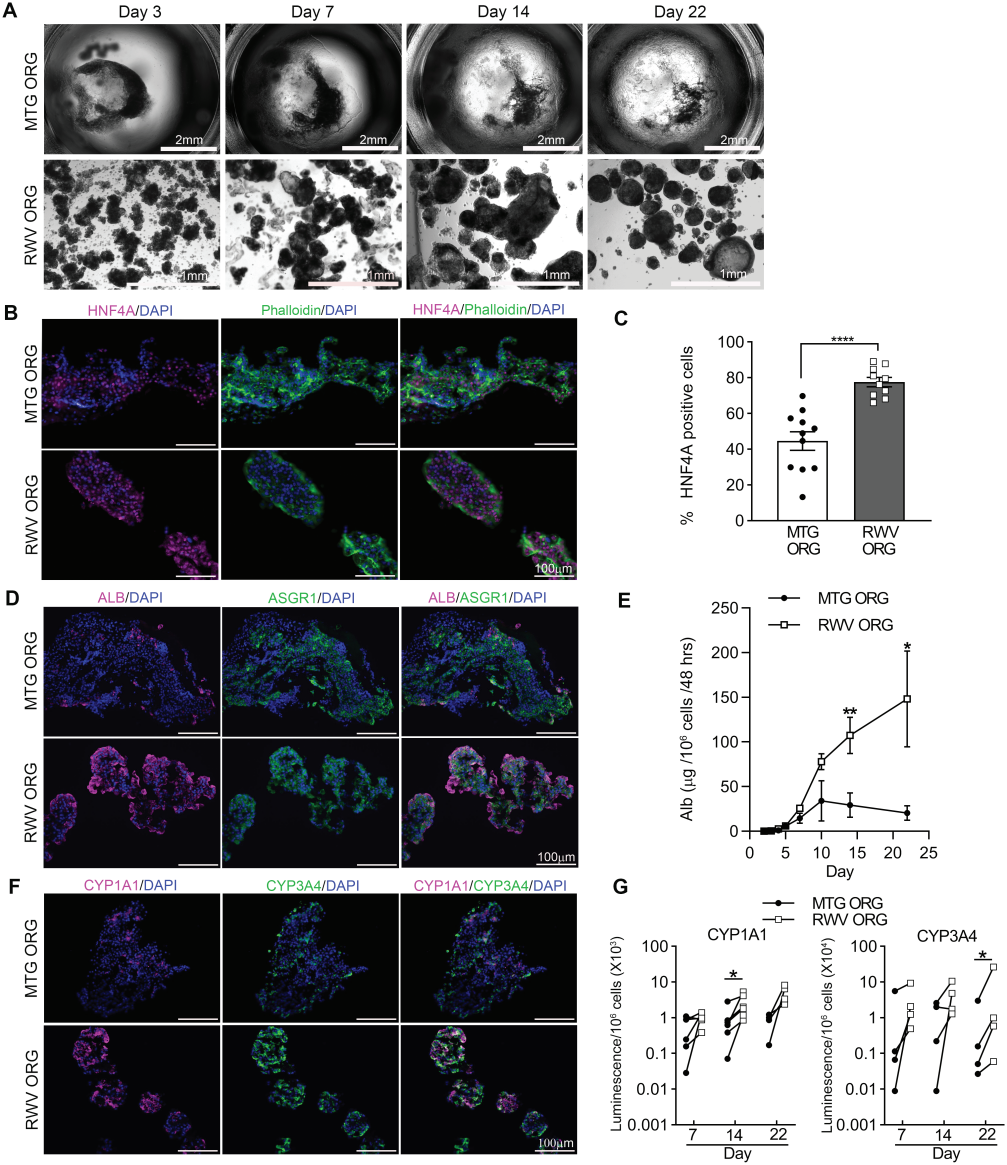
RWV ORGs exhibit sustained organoid structural integrity and superior hepatocyte-specific functions as compared to MTG ORG over long-term culture. (**A**) Bright-field images of MTG ORGs and RWV ORGs from day 3 to day 22 of organoid culture. Images are representative of 5 independent experiments. (**B**) Immunofluorescence staining of day 14 MTG ORGs and RWV ORGs showing HNF4A , phalloidin, and DAPI. Scale bar = 100 µm. Images are representative of 3 independent experiments. (**C**) Percentage of HNF4A^+^ cells in day 14 organoids. Number of HNF4A^+^ cells and total cells (DAPI^+^) were counted in each fluorescence image, and % of HNF4A^+^ cells calculated as (HNF4A^+^ cells/ total cells) * 100. Low-powered images (20× magnification) were taken of each condition from 3 independent experiments. Graphs show data from individual images and mean ± SEM; ****P < .0001 by 2-tailed Student’s *t* test. (**D**) Immunostaining of day 14 MTG ORGs and RWV ORGs showing ALB, ASGR1, and DAPI. Scale bar = 100 µm. (**E**) Albumin production by MTG ORGs and RWV ORGs through time. Data show mean ± SEM; *n* = 3-7 at each timepoint from independent experiments; *P < .05, **P < .01 by 2-tailed Student’s *t* test. (**F**) Immunostaining of day 14 MTG ORGs and RWV ORGs showing CYP1A1, CYP3A4, and DAPI. Scale bar = 100 µm. (**G**) CYP1A1 and CYP3A4 metabolic activities in MTG ORGs and RWV ORGs on days 7, 14, and 22. Data show paired results in 4-6 independent experiments (*n* = 4-6) at each timepoint; *P < .05 by paired *t* test.

To examine the durability of hepatic characteristics in RWV ORGs, we evaluated the long-term maintenance of organoid structure and hepatocyte-specific functions in MTG ORGs and RWV ORGs. By 3 days of culture, MTG ORG conditions typically showed a single curvilinear or doughnut-shaped organoid in each culture well. MTG ORGs compacted into more spherical forms around day 7, but thereafter, 3D structure was progressively more disorganized and became disintegrated by day 22 (Fig. 6A). In contrast, organoids formed within RWVs by day 3 of culture continued to maintain their spheroid structure to at least day 22. These observations suggest that the adhesion properties of cells within MTG ORGs change over time and contribute to the transitory nature of 3D organization. Conversely, the RWV environment promotes and sustains 3D cellular self-assembly, resulting in stable organoid structures through time.

We found that sustained organoid architecture correlated with greater long-term hepatic functions in RWV ORGs as compared to MTG ORGs. At 14 days of organoid culture, the percentages of HNF4A^+^ cells were 2-fold higher in RWV ORGs than in MTG ORGs (Fig. 6B, 6C). Albumin-positive cells were also significantly increased in RWV ORGs as compared with MTG ORGs (Fig. 6D). Importantly, RWV ORGs showed markedly higher albumin secretion per cell in longer-term culture (days 14 and 22) compared to MTG ORGs (Fig. 6E). Whereas MTG ORGs showed a gradual decline in albumin secretion after day 10, albumin production grew ever more robust in RWV ORGs and continued to increase at later timepoints. The rate of albumin production in RWV ORGs at day 22 (~150 μg/million cells/48 h) was comparable to the estimated rate of albumin production by human hepatocytes in vivo.^40^ In addition, at day 14, RWV ORGs demonstrated more CYP1A1^+^ and CYP3A4^+^ cells than MTG ORGs, indicating greater drug metabolic capabilities (Fig. 6F). Indeed, RWV ORGs showed significantly higher CYP1A1 and CYP3A4 activity per cell at days 14 and 22, respectively, as compared to MTG ORGs (Fig. 6G). These data indicate that transcriptomic evidence of increased hepatic function in RWV ORGs corresponded with sustained greater functional performance in long-term culture.

### RWV ORGs Express Many Mature Functional Genes At Levels Comparable to Adult Human Liver Tissue And Retain the Expression of Some Fetal Hepatoblast Markers

**Figure 7.**
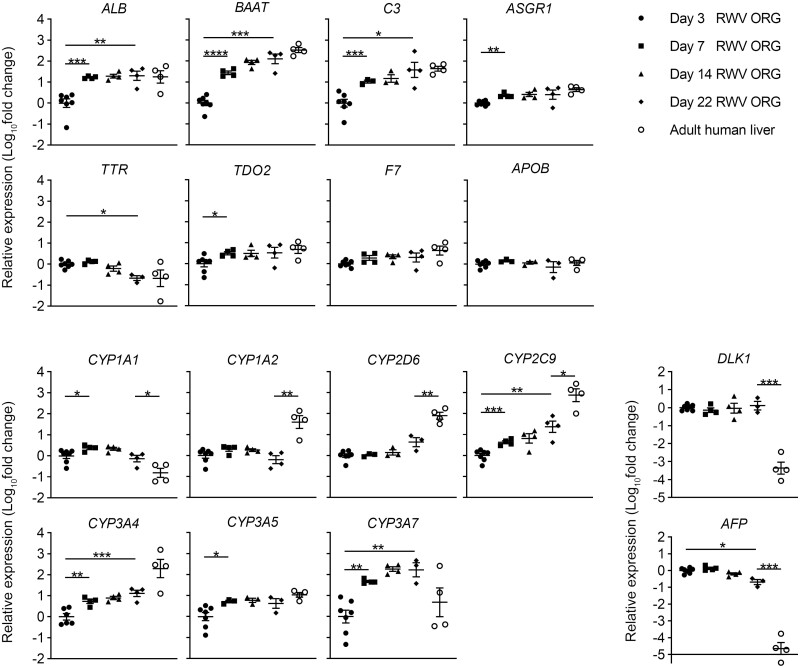
RWV ORGs demonstrate comparable expression of many mature functional genes with respect to adult human liver tissue, while still displaying some fetal markers. RNA was isolated from days 3, 7, 14, and 22 of RWV ORGs (*n* = 3-7 independent experiments at each timepoint) and liver tissue of 4 human donors with no known liver abnormalities. qRT-PCR analysis was performed to determine (**A**) hepatocyte-specific functional genes, (**B**) cytochrome P450 family genes, and (**C**) fetal hepatoblast marker genes. Data show independent samples and mean ± SEM; *n* = 3-7; *P < .05, **P < .01, ***P < .001, ****P < .0001 by Welch’s *t* test.

To investigate the maturity of RWV ORGs in comparison to adult primary human hepatocytes, we analyzed the gene expression of RWV ORGs at days 3, 7, 14, and 22 of culture, and liver tissue from 4 adult donors with no known liver abnormalities. We performed quantitative real-time reverse transcription PCR (qRT-PCR) using a gene panel that included *ALB, BAAT, C3, ASGR1, TTR, TDO2, F7,* and *APOB*. These genes represented a diverse cross-section of the hepatic secretome and metabolic functions significantly upregulated in RWV ORGs as compared to MTG ORGs (Fig. 2E, 2F). We found that on day 22 RWV ORGs expressed these functional genes at levels similar to adult human liver tissue (Fig. 7A). *ALB*, *BAAT*, and *C3* expression in RWV ORGs steadily increased through time from day 3 to day 22, so by day 22, expression of these genes in RWV ORGs were comparable to primary liver tissue. RWV ORGs expressed *ASGR1, TTR, TDO2, F7,* and *APOB* at levels similar to primary liver tissue as early as day 3-7 of culture. These findings suggest that RWV ORGs recapitulate many functional features of primary liver tissue with a high degree of fidelity.

Xenobiotic metabolism is a critical liver function. Some of the most important cytochrome P450 enzymes involved in drug metabolism are 1A1, 1A2, 2D6, 2C9, 3A4, and 3A5.^41^ We found that RWV ORGs expressed *CYP1A2*, *CYP2D6*, *CYP2C9*, and *CYP3A4* at lower levels than compared to primary liver tissue (Fig. 7B). *CYP2C9* and *CYP3A4* expression in RWV ORGs progressively increased from day 3 to day 22 culture, but the expression of these cytochromes remained lower in RWV ORGs than compared to normal adult liver. *CYP3A5* was the only cytochrome that showed comparable expression between day 22 RWV ORGs and primary liver tissue. Interestingly, *CYP1A1* and *CYP3A7,* both normally expressed in the fetal liver,^42,43^ were expressed more highly in day 22 RWV ORGs than in primary liver tissue. Other markers of fetal hepatoblasts, including *DLK1* and *AFP*,^44^ were also significantly more highly expressed in day 22 RWV ORGs than in adult liver tissue (Fig. 7C). These results suggest that RWV ORGs demonstrate many functional characteristics comparable to adult human liver while retaining some fetal features.

## Discussion

The results of our study suggest that RWV-facilitated 3D organization promotes hepatic lineage differentiation and improves organoid form and function compared to organoids generated with Matrigel. Our findings indicate that the way in which 3D cell assembly is induced has an impact on organoid phenotype.

Our findings show that organoid formation with iHECs promotes hepatic function, whereas organoid formation with iHEPs, which are cells already differentiated to the “hepatocyte” stage as monolayers on Matrigel, reduce hepatic function. This may be because iHEPs are not optimally differentiated as hepatocytes, due to Matrigel- and/or dimension-association factors, and has lost plasticity to further modify their phenotype upon incorporation into organoids. Indeed, our RNA-seq analysis showed that monolayer iHEPs displayed significantly depressed expression of a multitude of hepatic functional genes as compared to RWV ORGs that initiated organoid formation at the iHEC stage.

Our analysis indicated that TNFA signaling was upregulated in RWV ORGs, whereas the Wnt/β-catenin pathway was upregulated in MTG ORGs. Both TNFA^11^ and Wnt/β-catenin^45^ have been implicated in hepatic organoid generation and maintenance. Wnt/β-catenin has pleiotropic effects on multiple developmental pathways that are likely context dependent. Our results analyzing the effect of adding Matrigel to RWV ORGs suggest that Matrigel components promote alternative differentiation pathways associated with Wnt/β-catenin signaling, such as neural crest and kidney development. Thus, improved long-term hepatic function in RWV ORGs is likely the consequence of more uniform and streamlined hepatic differentiation within the 3D milieu created by RWVs. The stable 3D morphology of RWV ORGs may also contribute to their sustained greater hepatic function. Our RNA-seq analysis showed that multifactorial elements of the RWV environment were primarily responsible for supporting improved hepatic differentiation and function, and the absence of Matrigel-mediated adverse effects on hepatic lineage specification also played a role. Accordingly, our immunohistochemistry results indicated that there were more cells with hepatocyte features in RWV ORGs than in MTG ORGs. Future studies utilizing single-cell RNA-seq approaches can more thoroughly dissect the effect of culture conditions on the heterogeneity versus conformity of cell fate differentiation within organoids.

Our findings indicate that many hepatic functional markers were expressed in RWV ORGs at levels comparable to primary adult human liver tissue. However, mature hepatic drug-metabolizing cytochrome P450 enzymes (eg, *CYP1A2*, *CYP2D6*, *CYP2C19*, and *CYP3A4*) were expressed at significantly lower levels in RWV ORGs as compared to primary liver tissue, and RWV ORGs continued to express high levels of fetal hepatoblast markers (eg, DLK1 and AFP). These observations align with other studies that have shown that iPSC-derived hepatic cells remain “hepatocyte-like” and not fully mature.^28,44^ iPSC-derived hepatic cells may further mature after transplantation in vivo,^46^ but it remains unclear what in vivo factors are critical for inducing cells to achieve final maturation with incumbent full hepatocyte function. Ontogeny of mature hepatic cytochrome P450s, especially members of the CYP3A family, depends on the presence of gut microbiota,^47^ a feature not present in sterile cell culture. Factors elaborated by the gut microbiome may be the missing link to ultimate iPSC-derived hepatocyte differentiation and function in vitro, and RWV organoid culture can be used to examine this hypothesis in future studies.

Finally, to fully evaluate the potential of RWV ORGs for clinical therapeutic translation, we must further investigate their tumorigenic risk and efficacy in correcting liver dysfunction after in vivo engraftment. These types of studies will be a major focus of our research efforts going forward.

## Conclusion

We have shown that RWV culture is a robust approach to generate human iPSC-derived liver organoids with high and durable hepatic-specific functions. Matrigel is not necessary to produce 3D cell–cell structure and may have unintended alternative effects on cell differentiation. Producing organoids in RWVs is simple, high-throughput, and reduces the number of uncontrolled variables. Therefore, RWV-generated organoids are valuable models for investigating how multicellular 3D organization regulates cell form and function, and well-suited for development toward clinical-grade organoids for therapeutic applications.

## Supplementary Material

sxac090_suppl_Supplementary_Figure_S1

sxac090_suppl_Supplementary_Material

sxac090_suppl_Supplementary_Tables

## Data Availability

MINSEQE-compliant RNA-seq data are available through the Gene Expression Omnibus public repository (GSE222273).
