# Supplementary figures and images for: Self-Assembled Matrigel-Free iPSC-Derived Liver Organoids Demonstrate Wide-Ranging Highly Differentiated Liver Functions
Source: Stem Cells. 2022 Dec 27;41(2):126–39. doi: 10.1093/stmcls/sxac090 (PMC9982071; doi:10.1093/stmcls/sxac090)

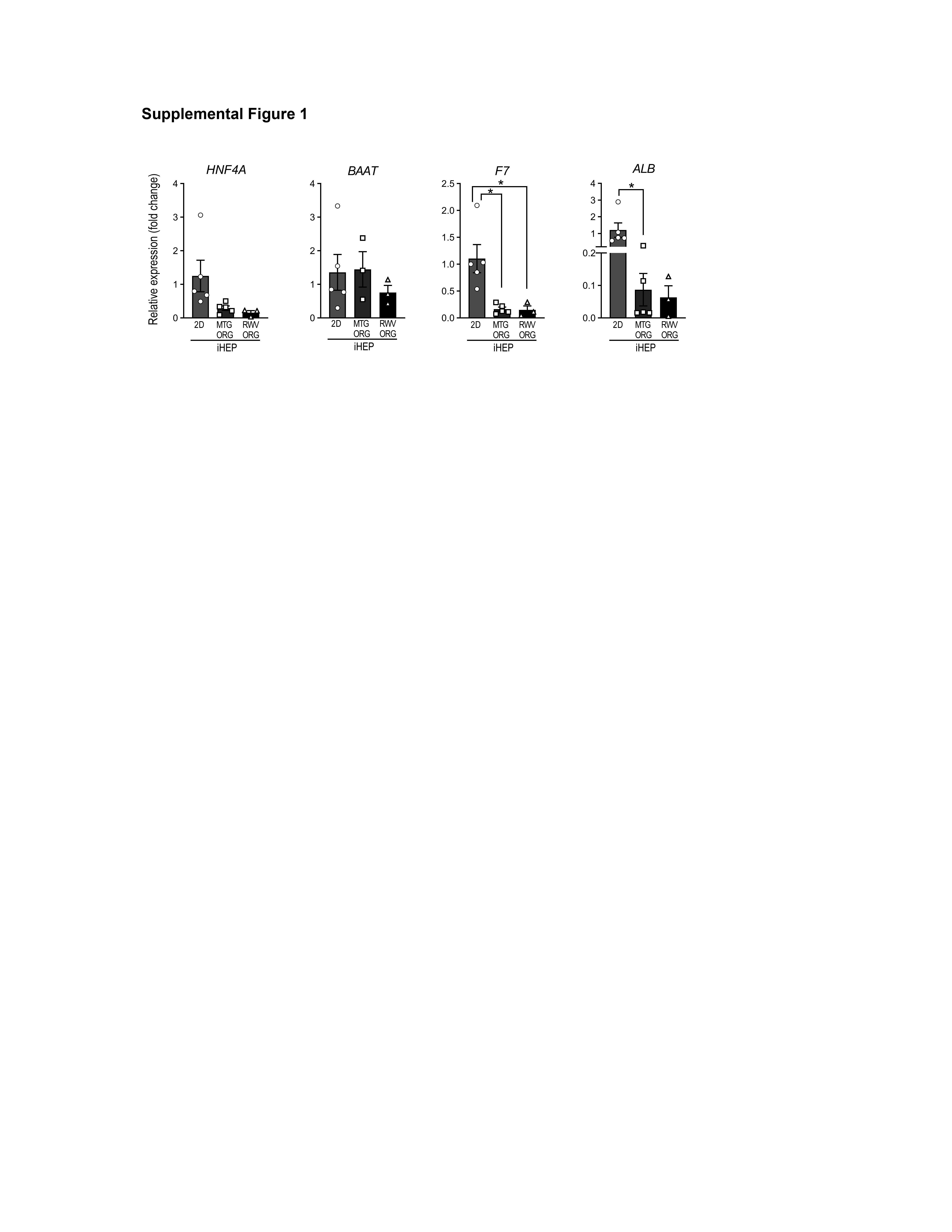

Supplement: sxac090_suppl_Supplementary_Figure_S1 [file sxac090_suppl_supplementary_figure_s1.jpeg]
