# Supplementary material for: Self-Assembled Matrigel-Free iPSC-Derived Liver Organoids Demonstrate Wide-Ranging Highly Differentiated Liver Functions
Source: Stem Cells. 2022 Dec 27;41(2):126–39. doi: 10.1093/stmcls/sxac090 (PMC9982071; doi:10.1093/stmcls/sxac090)
Supplement: sxac090_suppl_Supplementary_Material [file sxac090_suppl_supplementary_material.docx]

**Supplemental Experimental Procedures**

**Human induced pluripotent stem cell (iPSC) differentiation to induced hepatic endoderm cells (iHECs) and induced hepatocytes (iHEPs)**

A derivative of the reference human iPSC line Wt11c, which was extensively characterized to be pluripotent and genetically stable (Kreitzer et al., 2013), was obtained from Coriell Institute (Camden, NJ) and maintained with Cultrex basement membrane extract (R&D Systems, Minneapolis, MN) and StemFlex media (ThermoFisher Scientific, Waltham, MA). Human iPSC differentiation toward the hepatic lineage was performed according to published protocols (Peaslee et al., 2021; Si-Tayeb et al., 2010). For iHEC induction, 600,000 iPSCs per well were seeded in 6-well plates coated with 30-fold-diluted Matrigel (Corning Life Science, Glendale, AZ) in StemFlex media with 10μM Y27632 (Biogems, Westlake Village, CA). After 24 hours, media was exchanged to RPMI-basal-media consisting of RPMI 1640 (ThermoFisher Scientific) with 2% Gem21 serum-free supplement (Gemini Bio, West Sacremento, CA), 1x GlutaMax (Gibco, Waltham, MA), 1x NEAA (Gibco), 0.5 mM sodium butyrate (Sigma-Aldrich, Burlington, MA), 100ng/mL recombinant human Activin A (Peprotech Cranbury, NJ), 2µM doxycycline (MilliporeSigma, St. Louis, MO), and 10μM Y27632. On day 1 of induction, media was exchanged to RPMI-basal media plus 2% Knockout Serum Replacement (KSR) (Gibco), 3 mM CHIR-99021 (Selleckchem, Houston, TX), 50 nM PI-103 (Selleckchem), 10ng/mL recombinant human BMP4 (Peprotech), and 20 ng/mL FGF2 (Peprotech). On day 2, media was exchanged to RPMI-basal-media plus 1% KSR, 50nM PI-103, 10ng/mL BMP4 and 20 ng/mL FGF2. On day 3, media was exchanged to RPMI-basal media plus 0.2% KSR and 50 nM PI-103. For the first 7 days, cells were incubated at 20% oxygen, 5% CO_2_. On day 8, culture media were switched to IMDM-basal-media consisting of IMDM (Gibco) with 2% Gem21 serum-free supplement, 1x GlutaMax, 1x NEAA, 5μg/mL insulin (MilliporeSigma), 1ug/mL dexamethasone (Thomas Scientific, Swedesboro, NJ), 0.004% monothioglycerol (MilliporeSigma), 20ng/mL recombinant human BMP4, and 10 ng/mL FGF2. Cells were cultured in 5% oxygen and 5% CO_2_ for the next 5 days to generate iHECs. For iHEP induction, cells were continued under 5% oxygen and 5% CO_2_ incubation conditions in IMDM-basal media with 20ng/mL HGF for 5 days, followed by HCM media (Lonza, Walkersville, MD) containing 20ng/mL HGF and 20ng/mL OSM (Peprotech) incubated at 20% oxygen and 5% CO_2_ for another 5 days_._

**Organoid generation from iHEPs**

To generate iHEP-derived organoids, cells were digested by 50U/ml dispase (Corning) with 10μM Y27632 for 20min, following by 0.25% trypsin-EDTA (ThermoFisher Scientific) with 10uM Y27632 for 10min in 37 °C. Cell clumps were digested by Accutase (MilliporeSigma) for 10-20min until formation of single-cell suspensions. Cells were resuspended in Organoid Media, consisting of 50% HCM media (Lonza) and 50% EBM2 media (Lonza) with 40ng/mL HGF (PeproTech), 20ng/mL OSM (PeproTech), and 1μg/mL dexamethasone (Thomas Scientific) (Freyer et al., 2017). Hepatic organoids were generated by seeding cells either onto 24-well-plates coated with 2-fold-diluted Matrigel or into 10ml RWVs (Synthecon, Houston, TX) set to 10.5 rpm rotation. All organoid cultures were incubated in 37^o^C with 20% O_2_ and 5% CO_2_.

**Organoid collection**

Organoids generated on Matrigel were collected by adding 2ml Cultrex Organoid Harvesting Solution (R&D Systems) to the culture well and incubating at 2-8°C or on ice for 30–60min with moderate shaking. Organoids generated in RWVs were collected by aspirating the organoid suspension from the culture vessel.

**High-throughput RNA sequencing (RNA-seq) and analysis**

Total RNA was extracted using the RNeasy Plus Mini Kit (QIAGEN, Redwood City, CA), and RNA quantity and integrity were measured by 2100 Bioanalyzer (Agilent Technologies, Santa Clara, CA). Library construction, high-throughput sequencing, quality control, and sequence alignment were performed by Novogene (Sacramento, CA) per the company’s standard procedures. Briefly, 1μg of total RNA was used for sample preparations. Ribosomal RNA was removed by the Ribo-zero rRNA removal kit (Epicentre, Madison, WI) and the residual RNA cleaned by ethanol precipitation. Sequencing libraries were generated by NEBNext Ultra Directional RNA Library Prep Kit for Illumina (New England Biolabs, Ipswich, MA) following the manufacturer’s protocols. Library fragments were purified with AMPure XP system (Beckman Coulter, West Sacremento, CA) to obtain 250–300 bp cDNA fragments. Library quality was assessed by Agilent Technologies Bioanalyzer 2100, and high-throughput sequencing was performed on the Illumina HiSeq 4000 platform (San Diego, CA) using HiSeq 3000/4000 SBS Kit (300 cycles) with 20 million reads per sample. Raw reads in FASTQ format were processed by removing the adaptor reads or poly-N and low-quality reads. Error rate distribution and Q20, Q30, and GC content of the cleaned data were calculated. Quality-controlled reads were mapped to the human reference genome (hg19) with STAR alignment software (Dobin et al., 2013). Raw reads were normalized by DESeq2 algorithm (Love et al., 2014).

Downstream differential gene expression, clustering, and statistical analyses were performed using GeneSpring GX v14.9 (Agilent Technologies) on DESeq2-normalized log_2_-transformed counts. Dataset was filtered for genes that had log_2_-transformed expression of >0.01 in at least 2 samples. One-way ANOVA was used to compare >2 conditions with Benjamini-Hochberg multiple testing correction; threshold for significantly differentially-expressed genes was set to corrected p<0.05. Post-hoc Tukey’s HSD was applied to pairs of conditions. For some analyses, significantly differentially-expressed genes were further focused to genes with ≥2-fold difference between a pair of conditions.

Gene Set Enrichment Analysis (GSEA) and associated Leading Edge Analysis were performed using downloaded GSEA 4.2.3 software with gene set permutation, 1000 permutations, and selection of Cell Type Signature (C8), Hallmark (H), or Curated Canonical Pathways (C2 CP) gene set collections from the Molecular Signatures Database v7.5.1 (Liberzon et al., 2011; Subramanian et al., 2005).

Gene Ontology (GO) analysis was performed using the online tool GOrilla in the two-unranked-lists running mode (Ashburner et al., 2000; Eden et al., 2009). The target gene list included genes that were expressed ≥2-fold greater in RWV ORGs than in MTG ORGs as determined by moderate t-test with corrected p<0.05. The background gene list included all genes in the dataset with DESeq2-normalized log_2_-transformed expression >0.01 in at least 2 samples. Threshold p-value for significantly enriched GO terms was set to <0.001. GO analysis results were visualized using a modified version of previously published code (Bonnot et al., 2019).

We tested the addition of Rho-kinase (ROCK) inhibitor Y27632 at the start of organoid cultures because ROCK inhibition has been shown to improve stem cell-derived organoid formation in some settings (Ungrin et al., 2008; Yan et al., 2016). We found that addition of Y27632 had little effect on organoid formation efficiency from iHECs and had minimal impact on global gene expression as determined by RNA-seq (**Supplemental Tables 21** and **22**). Therefore, culture conditions with or without ROCK inhibitor were consolidated into the 3 main conditions as MTG ORG, RWV ORG, and RWV ORG + MTG for further RNA-seq analysis.

**Quantitative real-time reverse transcription PCR (qRT-PCR)**

Reverse transcription was carried out with 100ng of RNA using the High-Capacity cDNA Reverse Transcription Kit (ThermoFisher Scientific) following the manufacturer’s instructions. A total of 1μl of the resulting cDNA was added to a final 10μl mixture containing 5μl of 2x SYBR Green PCR Master Mix (ThermoFisher Scientific) and 3pmol oligonucleotide primers. Reactions were carried out in a 7300 Real-Time PCR System (ThermoFisher Scientific) using the thermal profile 50°C for 2min and 95°C for 10min, followed by 40 amplification cycles consisting of 95°C for 15s, 60°C for 30s, and 72°C for 30s. Samples were normalized to rRNA 18S internal standard. Relative quantification of gene expression was calculated by using the 2^ΔΔCt^ equation.

Sequences of primer pairs used in qRT-PCR are listed below.

| Gene name | Primer | Sequence 5'->3' | HPB ID |
| --- | --- | --- | --- |
| *18S* | Forward | GTGGAGCGATTTGTCTGGTT | NA^1^ |
|  | Reverse | CGCTGAGCCAGTCAGTGTAG |  |
| *HNF4A* | Forward | CACGGGCAAACACTACGGT | 71725338c1 |
|  | Reverse | TTGACCTTCGAGTGCTGATCC |  |
| *ALB* | Forward | CGTTCCCAAAGAGTTTAATGC | NA |
|  | Reverse | AAGCTGCGAAATCATCCATAAC |  |
| *BAAT* | Forward | AGTTGCCAGTGCTCCAAAGG | 189083864c3 |
|  | Reverse | GGCCCGAAATTCAAGCAGC |  |
| *F7* | Forward | AACCCCAAGGCCGAATTGT | 116805320c1 |
|  | Reverse | CGCGATCAGGTTCCTCCAG |  |
| *CYP1A1* | Forward | GGAGCTAGACACAGTGATTGGC | NA |
|  | Reverse | GGTGAAGGGGACGAAGGA |  |
| *CYP1A2* | Forward | CTTCGCTACCTGCCTAACCC | 73915099c3 |
|  | Reverse | GACTGTGTCAAATCCTGCTCC |  |
| *CYP2D6* | Forward | TGGCAAGGTCCTACGCTTC | 68509920c2 |
|  | Reverse | GCCACCACTATGCACAGGTT |  |
| *CYP2C9* | Forward | GGACAGAGACGACAAGCACA | NA |
|  | Reverse | CATCTGTGTAGGGCATGTGG |  |
| *CYP3A4* | Forward | TTCAACAGATGATCGACTCCCA | 16933531c2 |
|  | Reverse | TTGTGTCATAGGCAGCAAAAATG |  |
| *CYP3A5* | Forward | AATGTTTTGTCCTATCGTCAGGG | 4503231a1 |
|  | Reverse | AGACCTTCGATTTGTGAAGACAG |  |
| *CYP3A7* | Forward | AAACTTGGCCGTGGAAACCT | 262290931b1 |
|  | Reverse | CCCTTACGGAAGGACAAAGC |  |
| *APOB* | Forward | TGCTCCACTCACTTTACCGTC | 105990531c1 |
|  | Reverse | TAGCGTCCAGTGTGTACTGAC |  |
| *TTR* | Forward | ATGGCTTCTCATCGTCTGCT | NA |
|  | Reverse | TGTCATCAGCAGCCTTTCTG |  |
| *C3* | Forward | CTGTCCACGACTTCCCAGG | 115298677c2 |
|  | Reverse | CCCCTTTTCTGACTTGAACTCC |  |
| *TDO2* | Forward | TCCTCAGGCTATCACTACCTG | 375151559c2 |
|  | Reverse | ATCTTCGGTATCCAGTGTCGG |  |
| *ASGR1* | Forward | GAGAGAGACGTTCAGCAACTTC | 319655548c1 |
|  | Reverse | GGGACTCTAGCGACTTCATCTT |  |
| *DLK1* | Forward | AGGGTCCCCTTTGTGACCA | 290463105c2 |
|  | Reverse | GCAGGCCCGAACATCTCTATC |  |
| *AFP* | Forward | AGTGAGGACAAACTATTGGCCT | 4501988b3 |
|  | Reverse | ACACCAGGGTTTACTGGAGTC |  |

^1^Primers were designed using the online open-source Primer3 software (Koressaar et al., 2018), and others were from Harvard Primer Bank (HPB). Identification numbers (ID) for primer sequences obtained from HPB are listed. http://pga.mgh.harvard.edu/primerbank/citation.html. (Wang and Seed, 2003)

Primary human liver tissue RNA from 4 donors with no known liver abnormalities were obtained from commercial sources. Liver RNA from 3 donors were obtained from AMSBIO (Cambridge, MA): cat# R1234151-50, lot# B308121, male, age 44; cat# HR-314, male, age 65; cat# CR560055, lot# RN0000242E, female, age 51. An additional fourth donor liver RNA was obtained from ThermoFisher Scientific: cat# AM7960, lot# 2296016, female, age 59.

**CyQUANT cell proliferation assay**

Quantification of cell numbers was achieved by CyQUANT Cell Proliferation Assay kit (ThermoFisher Scientific) following the manufacturer's instructions.

**Albumin assay**

Albumin secretion was detected using a human-specific albumin enzyme-linked immunosorbent assay (ELISA) (Bethyl, Waltham, MA). Absorbances were measured using a multimodal microplate reader (Tecan, Zurich, Switzerland).

**Cytochrome P450 (CYP450) assay**

Cytochrome P450 activity was determined by P450-Glo CYP1A1 and CYP3A4 Assay kits (Promega, Madison, WI) according to the manufacturer’s protocol. Luciferase readings were taken with a GloMax-Multi+ Microplate Multimode Reader (Promega).

**Fluorescence immunostaining**

Organoids were fixed by 4% paraformaldehyde for 30-60min at room temperature, placed in 30% sucrose at 4°C overnight, followed by embedding in optimal cutting temperature (OCT) compound (Sakura, Torrance, CA) and freezing on dry ice. Embedded organoids were cut to 10μm cryosections and incubated in 10% donkey serum (Jackson ImmunoResearch, West Grove, PA), 0.1% triton X-100 (MilliporeSigma) in PBS for 1 hour in room temperature. Staining by primary antibodies was performed in 10% donkey serum, 0.1% triton X-100 in PBS overnight at 4°C. After three washes with PBS, organoid sections were incubated with the secondary antibody in 10% donkey serum, 0.1% triton X-100 in PBS for 1hr in room temperature. Following three washes, nuclei were stained by 5μg/ml Hoechst 33342 (Life technology, [Carlsbad, CA](https://www.google.com/search?client=firefox-b-1-e&q=Carlsbad&stick=H4sIAAAAAAAAAOPgE-LSz9U3MKmqSInPVeIAsYtMyvO0tLKTrfTzi9IT8zKrEksy8_NQOFYZqYkphaWJRSWpRcWLWDmcE4tyipMSU3awMu5iZ-JgAAC3PyAMVwAAAA&sa=X&ved=2ahUKEwjZ1sj2p8T4AhVUDkQIHSvhAloQmxMoAXoECHIQAw)) for 5-10mins. Coverslip were mounted with ProLong antifade solution (ThermoFisher) overnight.

Primary and secondary antibodies used are listed below.

**Primary antibodies**

| **Antibody** | **Vendor** | **Host** | **Class** | **Dilution** |
| --- | --- | --- | --- | --- |
| CYP1A1 (ab235185) | Abcam | Rabbit | Polyclonal | 1:100 |
| CYP3A4 (MA5-17064) | Thermo fisher | Mouse | Monoclonal | 1:100 |
| Albumin (A80-129A) | Bethyl | Goat | Polyclonal | 1:100 |
| HNF4A (sc374229) | Santa Cruz | Mouse | Monoclonal | 1:200 |
| Ki67 (RM-9106) | Epredia | Rabbit | Monoclonal | 1:100 |
| Claudin-2 (51-6100) | Thermo Fisher | Rabbit | Polyclonal | 1:100 |
| E-cadherin (610181) | BD Biosciences | Mouse | Monoclonal | 1:100 |
| ASGR1 (MAB4394) | R & D | Mouse | Monoclonal | 1:100 |
| Nephrin (NPHS1) (ABT331) | Millipore Sigma | Rabbit | Polyclonal | 1:100 |
| PODXL (393800) | Invitrogen | Mouse | Monoclonal | 1:100 |

**Secondary antibodies/reagents**

| **Antibody** | **Vendor** | **Dilution** |
| --- | --- | --- |
| Alexa Fluor 594-conjugated Donkey Anti-Mouse IgG (715-585-151) | Jackson ImmunoReasearch | 1:500 |
| Alexa Fluor 647-conjugated Donkey Anti-Rabbit IgG (711-605-152) | Jackson ImmunoReasearch | 1:500 |
| Alexa Fluor 594-conjugated Donkey Anti-Goat IgG (705-585-147) | Jackson ImmunoReasearch | 1:500 |
| Alexa Fluor 488-conjugated Donkey Anti-Mouse IgG (715-545-150) | Jackson ImmunoReasearch | 1:500 |
| Alexa Fluor 488 phalloidin (A12379) | Life technology | 1:400 |

**Supplemental References**

Ashburner, M., Ball, C.A., Blake, J.A., Botstein, D., Butler, H., Cherry, J.M., Davis, A.P., Dolinski, K., Dwight, S.S., Eppig, J.T., et al. (2000). Gene ontology: tool for the unification of biology. The Gene Ontology Consortium. Nat Genet *25*, 25-29. 10.1038/75556.

Bonnot, T., Gillard, M.B., and Nagel, D.H. (2019). A Simple Protocol for Informative Visualization of Enriched Gene Ontology Terms. Bio-protocol *9*, e3429. 10.21769/BioProtoc.3429.

Dobin, A., Davis, C.A., Schlesinger, F., Drenkow, J., Zaleski, C., Jha, S., Batut, P., Chaisson, M., and Gingeras, T.R. (2013). STAR: ultrafast universal RNA-seq aligner. Bioinformatics *29*, 15-21. 10.1093/bioinformatics/bts635.

Eden, E., Navon, R., Steinfeld, I., Lipson, D., and Yakhini, Z. (2009). GOrilla: a tool for discovery and visualization of enriched GO terms in ranked gene lists. BMC Bioinformatics *10*, 48. 10.1186/1471-2105-10-48.

Freyer, N., Greuel, S., Knospel, F., Strahl, N., Amini, L., Jacobs, F., Monshouwer, M., and Zeilinger, K. (2017). Effects of Co-Culture Media on Hepatic Differentiation of hiPSC with or without HUVEC Co-Culture. Int J Mol Sci *18*. 10.3390/ijms18081724.

Koressaar, T., Lepamets, M., Kaplinski, L., Raime, K., Andreson, R., and Remm, M. (2018). Primer3_masker: integrating masking of template sequence with primer design software. Bioinformatics *34*, 1937-1938. 10.1093/bioinformatics/bty036.

Kreitzer, F.R., Salomonis, N., Sheehan, A., Huang, M., Park, J.S., Spindler, M.J., Lizarraga, P., Weiss, W.A., So, P.L., and Conklin, B.R. (2013). A robust method to derive functional neural crest cells from human pluripotent stem cells. Am J Stem Cells *2*, 119-131.

Liberzon, A., Subramanian, A., Pinchback, R., Thorvaldsdottir, H., Tamayo, P., and Mesirov, J.P. (2011). Molecular signatures database (MSigDB) 3.0. Bioinformatics *27*, 1739-1740. 10.1093/bioinformatics/btr260.

Love, M.I., Huber, W., and Anders, S. (2014). Moderated estimation of fold change and dispersion for RNA-seq data with DESeq2. Genome Biol *15*, 550. 10.1186/s13059-014-0550-8.

Peaslee, C., Esteva-Font, C., Su, T., Munoz-Howell, A., Duwaerts, C.C., Liu, Z., Rao, S., Liu, K., Medina, M., Sneddon, J.B., et al. (2021). Doxycycline Significantly Enhances Induction of Induced Pluripotent Stem Cells to Endoderm by Enhancing Survival Through Protein Kinase B Phosphorylation. Hepatology *74*, 2102-2117. 10.1002/hep.31898.

Si-Tayeb, K., Noto, F.K., Nagaoka, M., Li, J., Battle, M.A., Duris, C., North, P.E., Dalton, S., and Duncan, S.A. (2010). Highly efficient generation of human hepatocyte-like cells from induced pluripotent stem cells. Hepatology *51*, 297-305. 10.1002/hep.23354.

Subramanian, A., Tamayo, P., Mootha, V.K., Mukherjee, S., Ebert, B.L., Gillette, M.A., Paulovich, A., Pomeroy, S.L., Golub, T.R., Lander, E.S., and Mesirov, J.P. (2005). Gene set enrichment analysis: a knowledge-based approach for interpreting genome-wide expression profiles. Proc Natl Acad Sci U S A *102*, 15545-15550. 10.1073/pnas.0506580102.

Ungrin, M.D., Joshi, C., Nica, A., Bauwens, C., and Zandstra, P.W. (2008). Reproducible, ultra high-throughput formation of multicellular organization from single cell suspension-derived human embryonic stem cell aggregates. PLoS One *3*, e1565. 10.1371/journal.pone.0001565.

Wang, X., and Seed, B. (2003). A PCR primer bank for quantitative gene expression analysis. Nucleic Acids Res *31*, e154.

Yan, Y., Song, L., Tsai, A.C., Ma, T., and Li, Y. (2016). Generation of Neural Progenitor Spheres from Human Pluripotent Stem Cells in a Suspension Bioreactor. Methods Mol Biol *1502*, 119-128. 10.1007/7651_2015_310.

**Supplemental Figure Legend**

**Supplemental Figure 1.**  **Organoids generated from induced hepatocytes (iHEPs) show reduced functional gene expression as compared to originating iHEP monolayers.** Gene expression of iHEP monolayers (iHEP 2D) and iHEP-derived organoids generated on Matrigel or within RWVs after 3 days of culture, represented as relative fold change compared to iHEP 2D. Data show independent biological samples and mean ± SEM; *p <0.05 by 2-tailed Student’s *t* test.
